# Next-Generation Sequencing Defines a Molecularly Confirmed ARPKD Core Within the Broader *PKHD1*-Associated Disease Spectrum

**DOI:** 10.3390/genes17020229

**Published:** 2026-02-11

**Authors:** Paloma Lapunzina-Soler, Amir Shabaka, Ramón Peces, Ángel Alonso, Emilio Cuesta, Rocío Mena, Laura Espinosa-Román, Marta Melgosa, Gema Fernández, Yolanda Muñoz-GᵃPorrero, Jair Tenorio-Castaño, Pablo Lapunzina, Julián Nevado

**Affiliations:** 1 Escuela de Doctorado, Universidad de Navarra, 31009 Pamplona, Spain; palomalapunzina@gmail.com; 2 Servicio de Nefrología de Adultos, Hospital Universitario La Paz, 28046 Madrid, Spain; amir.shabaka@salud.madrid.org (A.S.); rpecser@gmail.com (R.P.); gema.fernandezjuarez@salud.madrid.org (G.F.); 3 Servicio de Nefrología Infantil, Hospital Universitario La Paz, 28046 Madrid, Spain; aamelgar@salud.madrid.org (Á.A.); lespinosar@salud.madrid.org (L.E.-R.); marta.melgosa@salud.madrid.org (M.M.); 4 Servicio de Radiología, Hospital Universitario La Paz, IdiPAZ, Universidad Autónoma, 28046 Madrid, Spain; emilio.cuesta@salud.madrid.org; 5 Instituto de Genética Médica y Molecular, INGEMM, Hospital Universitario La Paz, 28046 Madrid, Spain; m.rocio.mena@gmail.com (R.M.); ymunozg@salud.madrid.org (Y.M.-G.); jaira.tenorio@salud.madrid.org (J.T.-C.); pablo.lapunzina@salud.madrid.org (P.L.); 6 CIBERER, Centro de Investigación Biomédica en Red de Enfermedades Raras, ISCIII, 28046 Madrid, Spain; 7 ITHACA—European Reference Network, 28046 Madrid, Spain; 8 Facultad HM de Ciencias de la Salud, Universidad Camilo José Cela, 28015 Madrid, Spain

**Keywords:** autosomal recessive polycystic kidney disease, Caroli disease, ductal plate malformation, fibrocystin/polyductin, *PKHD1*-assocaited disease spectrum, renal failure

## Abstract

**Background/Objectives**: Autosomal recessive polycystic kidney disease (ARPKD) is a severe ciliopathy caused by biallelic pathogenic variants in *PKHD1*, characterized by variable renal and hepatobiliary involvement. The widespread use of next-generation sequencing (NGS) has revealed a large number of rare *PKHD1* variants, creating major challenges in distinguishing molecularly confirmed ARPKD from a broader spectrum of *PKHD1*-associated disease. **Methods**: We performed an integrated clinical and molecular analysis of 68 individuals referred for suspected ARPKD. Using phase-aware and family-informed ACMG classification, patients were stratified into three genetically defined groups: 40 with molecularly confirmed ARPKD (biallelic pathogenic, likely pathogenic or segregation-supported VUS-LP variants *in trans*), 10 with biallelic *PKHD1* variants of uncertain pathogenicity, and 18 monoallelic carriers. Genotype–phenotype correlations were restricted to the molecularly confirmed ARPKD group. **Results**: Among the 40 molecularly confirmed ARPKD patients, 17 (42.5%) carried two loss-of-function (LoF) alleles, 16 (40%) carried one LoF allele, and 7 (17.5%) carried only non-LoF alleles. A strong allele-dose effect was observed. Neonatal or infantile onset occurred in 88% of LoF/LoF patients, compared with 56% of LoF/non-LoF and 29% of non-LoF/non-LoF individuals (*p* < 0.001). Progression to renal replacement therapy occurred in 65%, 31%, and 0% of patients (*p* = 0.002). In contrast, hepatobiliary disease was highly prevalent across all genotype classes and showed no significant association with LoF burden. **Conclusions**: Phase-aware and family-informed interpretation of *PKHD1* variants distinguishes a molecularly confirmed ARPKD core from a broader *PKHD1* variant spectrum. Within confirmed ARPKD, loss-of-function allele burden is the primary determinant of renal and perinatal severity, whereas hepatic disease is largely independent of truncating allele burden. These findings refine diagnosis, prognosis, and genetic counseling in the genomic era.

## 1. Introduction

Autosomal recessive polycystic kidney disease (ARPKD; MIM #263200) is a severe hepatorenal ciliopathy characterized by fusiform dilatation of the renal collecting ducts and progressive congenital hepatic fibrosis. Its estimated incidence ranges from 1:20,000 to 1:40,000 live births, although the true prevalence is likely underestimated due to clinical variability and overlap with other cystic kidney diseases [1,2,3]. Neonatal presentation typically includes bilateral nephromegaly, systemic hypertension, and respiratory compromise secondary to oligohydramnios, whereas long-term survivors frequently develop chronic kidney disease (CKD), portal hypertension, and recurrent cholangitis [4]. ARPKD is most commonly caused by biallelic variants in *PKHD1*, which encodes fibrocystin/polyductin, a cilia-associated protein essential for tubular and biliary architecture [5]. More than 700 pathogenic or likely pathogenic *PKHD1* variants have been reported so far, many of which are family-specific, complicating genotype–phenotype correlations [6]. In addition, biallelic pathogenic variants in *DZIP1L* have also been identified in a small subset of patients with ARPKD-like disease, further expanding the genetic spectrum and highlighting the role of primary cilia dysfunction in disease pathogenesis [7]. Despite comprehensive sequencing strategies, approximately 10–15% of clinically suspected ARPKD cases remain genetically unresolved, suggesting the involvement of additional genes, undetected structural variants, or noncoding variants not captured by standard pipelines [8]. In fact, the allelic architecture of *PKHD1* is highly complex. The gene spans more than 85 kb and harbors hundreds of rare missense and truncating variants, most of which are private or extremely infrequent in population databases. As a result, variant interpretation is challenging, and reliance on in silico prediction or database annotation alone leads to substantial overestimation of disease-causing alleles. Recent population-based studies have highlighted a striking discrepancy between predicted ARPKD prevalence based on rare *PKHD1* variants and the much lower clinically observed prevalence, underscoring the need for phase-aware and family-based variant interpretation [9,10].

Advances in massive paralleled sequencing (NGS), including multigene panels, whole-exome sequencing (WES), and whole-genome sequencing (WGS), have markedly improved diagnostic yield in cystic kidney diseases. These approaches enable the simultaneous analysis of *PKHD1*, *DZIP1L*, and other potential genes associated with phenocopies, such as early-onset autosomal dominant polycystic kidney disease (ADPKD), nephronophthisis, Joubert spectrum disorders, and *HNF1B*-related disease [11,12,13]. Accurate distinction between these entities is clinically relevant for prognosis, recurrence risk, and clinical management, particularly in infants, in whom imaging findings may be nonspecific and genetic testing is essential. However, this has also blurred the boundary between true ARPKD and a broader *PKHD1*-associated disease spectrum driven by variants of uncertain pathogenicity. In particular, the role of missense and hypomorphic alleles, and the distinction between pathogenic variants and variants of uncertain significance (VUS), remain major sources of diagnostic ambiguity.

Despite extensive research, contemporary real-world cohorts integrating systematic renal and hepatic phenotyping with broad genomic testing remain limited. Consequently, the mechanisms underlying the marked clinical heterogeneity of ARPKD are still incompletely understood, with most genotype–phenotype correlations restricted to the association of biallelic truncating *PKHD1* variants with severe disease [14,15]. As improvements in neonatal care have increased survival, there is a growing need to characterize the expanding phenotypic spectrum, including milder, atypical, and late-onset presentations, which have been comparatively underreported.

In this study, we present an integrated clinical and molecular characterization of a cohort of pediatric- and adult-onset patients with suspected ARPKD evaluated using standardized imaging protocols and contemporary NGS-based diagnostic workflows. Our aims were to (i) define a molecularly confirmed ARPKD core within the broader *PKHD1* variant spectrum, and (ii) determine how loss-of-function (LoF) allele burden shapes renal and hepatic disease severity. We describe the clinical, biochemical, imaging, and molecular features of 50 individuals with biallelic *PKHD1* variants, as well as 18 additional subjects carrying monoallelic pathogenic or likely pathogenic *PKHD1* variants. Collectively, our data delineate renal glomerular and tubular dysfunction, integrate molecular and imaging findings, and provide prognostic insights into the contemporary spectrum of ARPKD.

## 2. Materials and Methods

### 2.1. Study Design and Patient Recruitment

We conducted an observational, prospective cohort study including individuals evaluated for suspected ARPKD between 2018 and 2025 at Hospital Universitario La Paz (Madrid, Spain). Patients were identified through the Departments of Nephrology (adults and children), Neonatology, Hepatology, and Clinical Genetics. Inclusion criteria included (i) bilateral renal involvement compatible with ARPKD on ultrasound or MRI (increased echogenicity, poor corticomedullary differentiation, fusiform dilatation of collecting ducts, or nephromegaly), or (ii) clinical signs consistent with congenital hepatic fibrosis or portal hypertension, or (iii) referral for ARPKD-targeted genetic testing. Exclusion criteria included confirmed diagnosis of alternative cystic kidney disorders prior to evaluation. Demographic and clinical variables were collected from electronic medical records. Informed consent was obtained from all participants and/or their legal guardians at the time of genetic testing.

### 2.2. Clinical and Imaging Evaluation

Renal assessment included serial measurements of kidney size, Doppler-derived resistive indices, corticomedullary differentiation, and the presence of medullary ectasia or microcysts. Estimated glomerular filtration rate (eGFR) was calculated using age-appropriate formulas (Schwartz or CKD-EPI) [15,16,17,18]. Blood pressure percentiles were interpreted according to the 2017 AAP pediatric guideline [19]. Hepatic involvement was evaluated through liver ultrasound, elastography when available, presence of portal hypertension (splenomegaly, thrombocytopenia, collateral circulation), and history of cholangitis. Standardized imaging protocols were applied to all patients following institutional and international guidelines for pediatric hepatorenal ciliopathies.

### 2.3. Genetic Testing and Variant Interpretation

Genomic DNA was extracted from peripheral blood using standard procedures. Genetic testing was performed by targeted next-generation sequencing (NGS) using NEFROseq^®^ panel (v1.1 and v2.1), a custom designed panel covering 230 and 392 genes, including either *PKHD1* and other genes associated with ARPKD phenocopies and dominant polycystic kidney and liver disease (e.g., *PKD1*, *PKD2*, *DZIP1L*, *IFT140*, *ALG8*, *ALG9*, *HNF1B*, *NPHP1–13*). Target enrichment was carried out with SeqCap EZ technology (Roche NimbleGen, Madison, WI, USA) and sequencing was performed on Illumina NextSeq 500 (2018–2019) or NovaSeq 6000 (2019–2025) platforms (Illumina, San Diego, CA, USA), according to manufacturer and laboratory protocols.

Bioinformatic analyses were performed in house following a standardized workflow. Reads were quality-trimmed (Trimmomatic v0.32), aligned to the GRCh37/hg19 reference genome (BWA-MEM), and processed for duplicate marking and quality metrics (Picard-tools v1.27), with additional post-processing and coverage assessment using SAMtools (v0.1.19) and BEDTools (v2.26.0). Single-nucleotide variants (SNVs) and small indels were called using a GATK-based pipeline (GATK v3.3-0; HaplotypeCaller) and/or Illumina Variant Caller v2.1. Variants were annotated and prioritized using ClinVar, dbSNP (v138), dbNSFP (v3.0), dbscSNV (v1.1), LOVD v3.0, VarSome v13.14.1, UniProt, gnomAD, and an in-house curated database, integrating in silico prediction scores (e.g., SIFT v6.2.1., PolyPhen-2 v2.2.3, MutationAssessor r4/v4, FATHMM v2.3, GERP++, PhyloP100way, and CADD v1.3). Population allele frequencies were evaluated using gnomAD v4.1., the 1000 Genomes Project, the Spanish Exome Variant Project, and NHLBI ESP6500 (ESP6500SI-V2-SSA137); variants with minor allele frequency (MAF) ≥ 1% in population datasets were generally not prioritized for ARPKD unless specific evidence supported pathogenicity. Copy-number variants (CNVs) were assessed from targeted sequencing data using XHMM (v1.2.3) and/or ExomeDepth v1.1.16, complemented by LACON v1.2 and Manta v1.5.0 when applicable. Variant interpretation followed ACMG–AMP guidelines [20]. For each *PKHD1* variant, both the qualitative ACMG category (pathogenic, likely pathogenic, VUS, likely benign, benign) and an internal quantitative ACMG scoring record were documented.

Phase (*in trans* vs. *in cis*) was established whenever parental samples were available. The ACMG criterion PM3 (*in trans* with a pathogenic variant in a recessive disorder) was applied only when segregation testing demonstrated that the two *PKHD1* variants were inherited from different parents. Apparently, de novo alleles were evaluated only in families with both parents tested and the variant absent in parental DNA, allowing application of PS2 when appropriate.

For downstream analyses, molecularly confirmed ARPKD was defined a priori as the presence of two *PKHD1* pathogenic/likely pathogenic variants in trans, or one P/LP variant in trans with one VUS with strong supporting evidence compatible with likely pathogenicity (VUS-LP) (4 cases). Individuals with at least one VUS not meeting these criteria were classified as having *PKHD1*-associated disease of uncertain molecular causality and were excluded from primary genotype–phenotype correlation analyses. Based on molecular results, patients were categorized as (a) molecularly confirmed ARPKD (biallelic disease-causing *PKHD1* variants *in trans*), (b) biallelic *PKHD1* variants of uncertain pathogenicity, or (c) monoallelic *PKHD1* carriers.

Predicted functional consequences were grouped as loss-of-function (LoF; nonsense, frameshift, canonical splice-site) or non-LoF (missense and in-frame), enabling stratification of allelic functional burden. In this study, the term hypomorphic is used in a strict functional sense and does not correspond to ACMG categories; hypomorphic variants were defined as alleles with demonstrated or strongly supported residual *PKHD1* function based on (i) segregation with milder disease in multiple affected individuals, (ii) recurrence in clinically attenuated ARPKD phenotypes, and/or (iii) published functional or transcript-level evidence. Variants classified as VUS or VUS-LB without such supporting evidence were not considered hypomorphic and were treated as molecularly unconfirmed.

#### Sanger Sequencing

*PKHD1* variant screening for coding sequences and intron/exon boundaries for specific exons was performed by direct sequencing. PCR conditions and primers (designed with the help of Primer3 plus v04.0 Software) were available upon request. PCR products were sequenced using Bright-Dye Terminator cycle kit (N imaging, Nijmegen, The Netherlands) and run on an ABI3730XL Sequencer (ThermoFisher, Waltham, MA, USA).

### 2.4. Outcomes and Follow-Up

Primary outcomes encompassed longitudinal renal function, progression of CKD stage, need for renal replacement therapy or transplantation, hepatic complications (portal hypertension and cholangitis), and all-cause mortality. Secondary outcomes included development of hypertension, growth metrics, and hospitalization rates. Follow-up data were updated as of 11 November 2025.

### 2.5. Statistical Analysis

Continuous variables were summarized as median or mean ± SD. Genotype–phenotype correlations were restricted to molecularly confirmed ARPKD. Categorical variables were compared across LoF/LoF, LoF/non-LoF, and non-LoF/non-LoF groups using Fisher’s exact test or χ^2^ test, with *p* < 0.05 considered significant. Analyses were performed using SPSS (v28; IBM, Armonk, NY, USA).

### 2.6. Ethical Considerations

Written informed consent was obtained from parents or legal guardians for all minors included, for NGS study. All procedures followed the principles of the Declaration of Helsinki. The study was made under protocols (C-GEN-004, 22 January 2016, and C-GEN-013, 25 April 2025) approved by the Institutional Review Board/Ethics Committee of Hospital Universitario La Paz

## 3. Results

### 3.1. Clinical Outcomes Cohort Characterization

**Figure 1:**
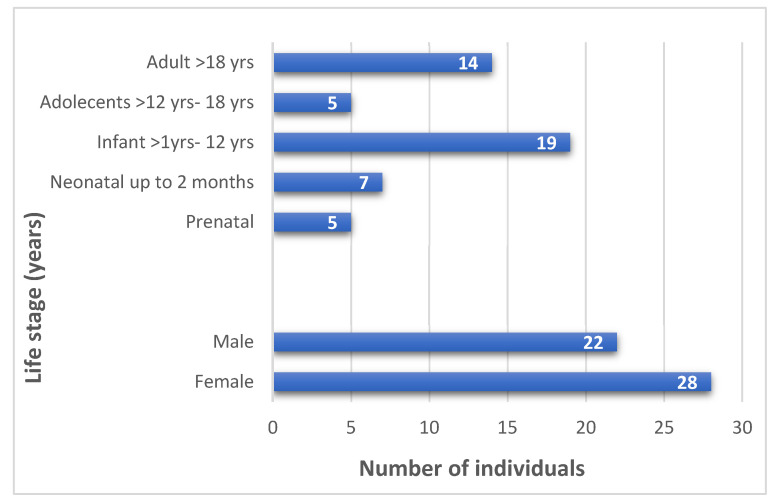
Age (years), sex, and life-stage distribution in the cohort.

Sixty-eight patients with a clinical suspicion of ARPKD were initially screened, of whom 50 showed biallelic variants in *PKHD1* were analyzed in this study. The median age at diagnosis was 24.8 years (range: prenatal up to 73 years), and 28 patients (56%) were female. Most individuals in the cohort were children (31/50, 63%; Figure 1).

This cohort represents a clinically heterogeneous patho-physiologically coherent spectrum of autosomal recessive polycystic kidney disease (ARPKD). It encompasses both severe prenatal, neonatal, and infantile presentations and attenuated adolescent- or adult-onset forms, which are increasingly recognized as part of the phenotypic continuum associated with *PKHD1*. Among adult patients, we have also identified a family with two affected members presenting with a markedly late disease onset, in the sixth decade of life (see below). Formal ancestry inference was not available for all participants and is acknowledged as a limitation. The cohort consisted predominantly of individuals of Eu-ropean ancestry, reflecting the referral population of our center, but two Hispanic pedi-gree.

At initial evaluation, 44% of patients (n = 22) exhibited systemic hypertension, and 30% (n = 15) showed impaired renal function (estimated glomerular filtration rate [eGFR] < 90 mL/min/1.73 m^2^; see Table 1). Prenatal abnormalities were detected in 24% of cases (n = 12), including oligohydramnios in 50% (6/12) and bilateral enlarged hyperechogenic kid-neys in 41.7% (5/12); all of these patients subsequently developed pulmonary hyperten-sion. Hepatic involvement was documented in 24 of 50 individuals (48%). Within this subgroup, hepatosplenomegaly was observed in 13 patients (54%), suspected Caroli disease in 24 patients (100%), and imaging findings consistent with congenital hepatic fibrosis in 13 patients (54%), among other manifestations (Table 1).

### 3.2. Imaging Characterization

Ultrasound revealed nephromegaly in 7/50 patients (14%), increased cortical echo-genicity in 13/50 (26%) patients, and loss of corticomedullary differentiation in 6/50 (12%) patients. Calyceal ectasia was shown in 9/50 (18%) patients, cortical and medullary cysts, some containing hemorrhagic/proteinaceous material, were shown in 13/50 (26%) patients, multiple bilateral cysts were shown in 10/50 (20%) patients, or simple cysts were shown in 6/50 (12%) patients (Table 1). Normal ultrasound findings were observed in only 4/50 (8%) individuals. MRI, when performed, was shown in most patients, fusiform dilatation of collecting ducts was shown in 24/50 (48%) patients, splenomegaly in 13/50 (26%) patients, and hepatic fibrosis in 13/50 (26%) patients (Table 1).

#### Organ-Specific Findings

- Renal involvement

Renal involvement ranged from severe neonatal ARPKD with early kidney failure to mild cystic disease with preserved function. Overall, 7/50 (14%) patients required renal replacement therapy and 7/50 (14%) underwent kidney transplantation. In contrast to historical series reporting high early mortality and frequent progression to end-stage kidney disease [21], most patients in our cohort were alive at last follow-up (45/50 with available outcome data, 90%) and many adults maintained moderate or only mildly impaired renal function. Most of the patient’s exhibited bilateral cystic kidney disease with diffuse cortico-medullary involvement, early-onset hypertension, and frequently requiring chronic antihypertensive therapy and progressive chronic kidney disease (CKD), with a substantial proportion reaching advanced stages (G3–G5). Multiple patients required renal replacement therapy, including chronic dialysis and kidney transplantation. Longitudinal imaging showed two distinct renal trajectories. In patients with early-onset disease, kidneys were initially enlarged and echogenic but frequently evolved toward progressive parenchymal loss and volume reduction over time. By contrast, adult-onset and hypomorphic genotypes often showed stable or slowly declining renal volumes with medullary-predominant cystic changes, as illustrated in the adult-onset family (see below).

- Hepatic involvement

Hepatobiliary disease was prominent: at least 37/50 (74%) of patients had hepatic involvement and 23/50 (46%) showed congenital hepatic fibrosis and/or Caroli-type changes. Portal hypertension with varices was documented in 17/50 (34%) patients, and 4/50 (8%) required liver transplantation. These data, in a survivor-enriched cohort, underscore that hepatobiliary morbidity becomes a major long-term problem in *PKHD1*-related disease, in line with recent genotype-phenotype studies highlighting progressive portal hypertension in childhood and adolescence [21,22,23]. A novel observation in our cohort is that liver manifestations were sometimes the earliest detectable abnormality, even in individuals with relatively preserved renal function. This supports emerging evidence that some *PKHD1* genotypes may produce a liver-predominant phenotype, broadening the recognized clinical spectrum of ARPKD.

- Pulmonary involvement and early mortality

Infantile and neonatal patients frequently showed pulmonary hypoplasia, severe respiratory distress, and early neonatal death. This pattern is consistent with severe prenatal ARPKD, secondary to oligohydramnios and restrictive thoracic development.

- Transplantation and therapeutic burden

A substantial proportion of patients required kidney transplantation, liver transplantation, and combined liver–kidney transplantation; chronic management included widespread use of antihypertensive drugs, endoscopic and medical management of portal hypertension, and recurrent antibiotic courses for biliary infections.

### 3.3. Molecular Classification of the Cohort

Among the 68 individuals evaluated for suspected ARPKD, 50 carried two or more *PKHD1* variants, whereas 18 were mono-allelic carriers.

**Figure 2:**
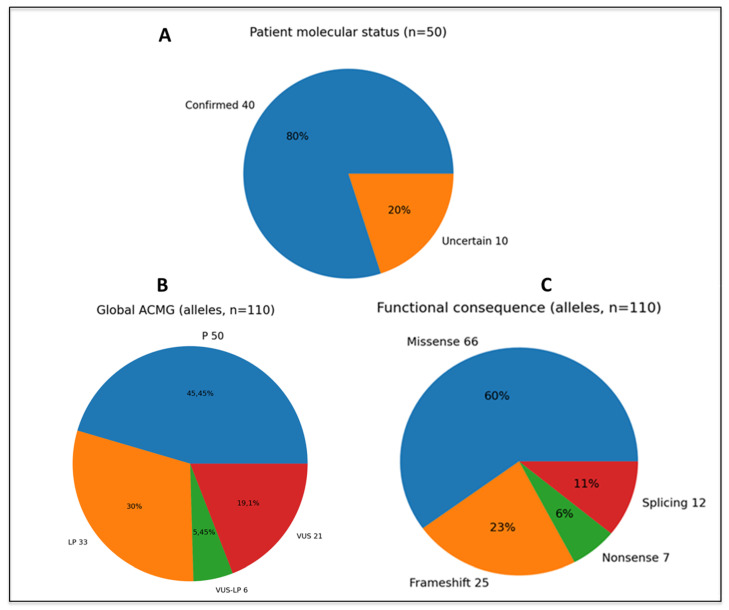
Distribution of *PKHD1* variants and biallelic genotypes after ACMG reclassification incorporating phase and segregation. (**A**) ARPKD Patient´s molecular status. (**B**) ACMG *PKHD1* variants final classification. (**C**) *PKHD1* variants type. ACMG, American College of Medical Geneticists; VUS, Variants of Unknown Significance; P, pathogenic; LP, likely pathogenic.

All *PKHD1* variants identified in the 50 patients with two or more *PKHD1* alleles were curated at the variant level and classified according to integrated ACMG/AMP guidelines; population frequency, in silico predictions, literature evidence, database annotations, and critically, segregation and parental testing whenever available. Incorporation of phase (*in trans*) and apparently *de novo* allele status enabled application of the ACMG criteria PM3 and PS2, respectively, leading to refinement of variant pathogenicity compared with database-only annotation (Table 2). Extended genetic analysis is shown in Table S1. Based on our clinical experience, variants established as VUS–LP (4 or 5 pathogenic points in ACMG) were also considered disease causing when present *in trans* with a P or LP *PKHD1* variant, and it was supported by segregation evidence (PM3); therefore, these cases were also included in the molecularly confirmed ARPKD group. Using this definition, 40 of 50 (80%) biallelic patients met criteria for molecularly confirmed ARPKD (see Table 2 and Figure 2A), while 10 patients (20%) carried at least one VUS–LB, likely benign, or conflicting variant and were classified as having an expanded *PKHD1*-associated disease spectrum (Table 2 and Figure 2A).

In addition, no pathogenic variants in *DZIP1L* were identified. However, 27 additional patients harbored variants in other genes associated with ARPKD phenocopies, including early-onset autosomal dominant polycystic kidney disease (*PKD1*, *PKD2*), nephronophthisis (*NPHP1–NPHP13*), and *HNF1B*-related disease, which were not included in the study.

All *PKHD1* variants were curated at the variant level and classified according to ACMG/AMP into 50 pathogenic variants (P, 50; 44.54%), 33 likely pathogenic variants (LP, 33; 27.27%), VUS-LP (6/31; 35.48%), and 21 variants of uncertain significance (VUS, 21; 28.18% (see Figure 2B). The variants by type are also described in Figure 2C.

#### 3.3.1. Functional Distribution of PKHD1 Alleles

Variants were subsequently grouped by predicted molecular-functional consequence. Among all 110 alleles, loss-of-function (LoF) variants were defined as frameshift, non-sense, or canonical splice-site variants fulfilling PVS1 criteria. Non-LoF variants included missense and in-frame variants across the ACMG spectrum (pathogenic, likely pathogenic, hypomorphic, and VUS). Using this functional classification, the distribution of biallelic genotypes in the cohort is shown in Table 3.

The variant spectrum was dominated by the known frameshift hotspots c.5895dupA (8/110 alleles; 7.3%) and c.9689delA (7/110; 6.4%), both previously reported as recurrent ARPKD pathogenic variants and potential founder alleles in specific populations (ClinVar; https://www.ncbi.nlm.nih.gov/clinvar/ (accessed on 20 December 2025), e.g., in our population). In our series, these “truncating” alleles were usually combined with a missense or splice-site variants rather than with a second truncating allele. Other frequent recurrent alleles were c.5134G>C (3/110; 2.7%), c.4870C>T (3/110; 1.8%), and c.6992T>A (2/110; 2.73%).

#### 3.3.2. Monoallelic Carriers

In addition to the 50 individuals with two or more *PKHD1* variants, we evaluated 18 subjects in whom only a single pathogenic or likely pathogenic *PKHD1* allele was detected (see Table S1). These individuals were not considered affected by ARPKD but were analyzed separately as monoallelic *PKHD1* carriers.

Several of these carriers exhibited mild renal or hepatobiliary imaging findings, including cortical microcysts, isolated renal cysts (Bosniak II) [24], biliary ectasia, or simple hepatic cysts. Such abnormalities are increasingly recognized in obligate heterozygotes for ARPKD and are consistent with previous reports describing subtle hepatorenal changes in *PKHD1* carriers (e.g., [25]). Importantly, however, none of the monoallelic carriers in our cohort showed neonatal ARPKD, congenital hepatic fibrosis, portal hypertension, or early-onset kidney failure, and none required renal replacement therapy for *PKHD1*-related disease.

These findings should not be interpreted as evidence that a single *PKHD1* variant causes ARPKD. Instead, they reflect either (i) reduced penetrance and subclinical expressivity in obligate carriers, or (ii) the presence of an undetected second allele, such as a deep intronic, regulatory, or structural variant not captured by current sequencing approaches. In a gene as large and complex as *PKHD1*, this possibility is well recognized and has been documented in multiple ARPKD cohorts. Crucially, monoallelic carriers must not be conflated with patients harboring two hypomorphic alleles. Hypomorphic genotypes represent biallelic combinations in which both variants retain partial function but together reduce fibrocystin activity below a pathogenic threshold. By contrast, monoallelic carriers retain one fully functional allele and therefore do not develop classical ARPKD. Consistent with this, the clinical manifestations observed in our heterozygous carriers were mild, non-progressive, and qualitatively distinct from those of molecularly confirmed ARPKD patients.

Together, these data reinforce the need to distinguish three entities: true ARPKD caused by biallelic pathogenic or functionally supported hypomorphic variants, an expanded *PKHD1*-associated disease spectrum driven by uncertain alleles, and monoallelic carrier states with minimal or subclinical manifestations. Failure to make this distinction is a major source of diagnostic confusion in the NGS era and a key driver of the apparent discrepancy between predicted and observed ARPKD prevalence.

#### 3.3.3. Whole-Cohort’s Allele Distribution

**Figure 3:**
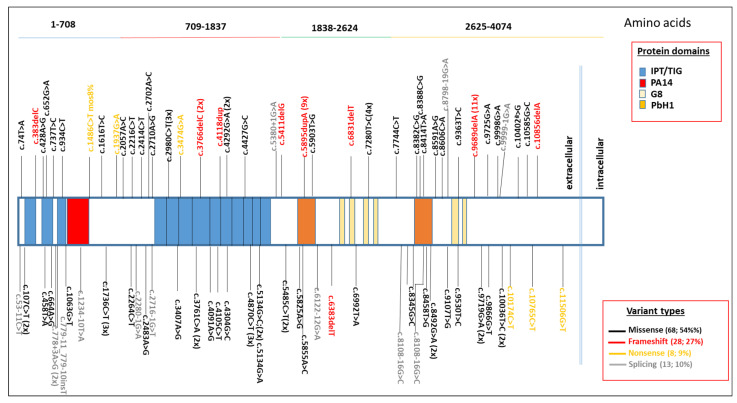
Distribution of all the detected *PKHD1* variants observed across the different protein domains and the variant type (missense, frameshift, nonsense, and splicing).

Overall, in our study we identified 117 *PKHD1* variants across all individuals, irrespective of zygosity or patient-level grouping, which are schematized in the Figure 3.

### 3.4. Genotype-Phenotype Correlations

To avoid confounding by variants of uncertain significance, genotype–phenotype correlations were performed using only the 40 patients with molecularly confirmed ARPKD, defined as above, and including cases with VUS-LP, all of them, in trans with a P/LP *PKHD1* variant. Patients were stratified into LoF/LoF (n = 17), LoF/non-LoF (n = 16), and non-LoF/non-LoF (n = 7) groups. A strong and graded association was observed between LoF allele burden and renal and perinatal severity (Table 4). Neonatal or infantile onset occurred in 15/17 of LoF/LoF patients, compared with 9/16 of LoF/non-LoF and 2/7 of non-LoF/non-LoF individuals (*p* < 0.001). Conversely, childhood or adult onset was most frequent among patients lacking truncating alleles (5/7 of non-LoF/non-LoF individuals; *p* < 0.001; Table 4).

Progression to end-stage kidney disease requiring renal replacement therapy (RRT) followed the same gradient. A total of 11 out of 17 LoF/LoF patients (65%) required dialysis or transplantation, compared with 5 out of 16 (31%) in the LoF/missense group, while no patient with two missense alleles progressed to RRT during follow-up. Finally, perinatal complications, including pulmonary hypoplasia, neonatal respiratory failure, or early mortality, were also most frequent in the LoF/LoF group and were rare in patients with biallelic non-LoF variants. Among patients who progressed to renal replacement therapy, the median age at initiation was markedly genotype dependent: 1.2 years (IQR 0.4–3.6) in the LoF/LoF group, 14.8 years (IQR 6.2–28.5) in the LoF/non-LoF group, and no events in the non-LoF/non-LoF group during follow-up. In contrast to renal outcomes, hepatic involvement was highly prevalent across all genetic classes, including congenital hepatic fibrosis, portal hypertension, and biliary abnormalities, and did not show a strong correlation with *PKHD1* functional class (see Table 4). In addition, no significant differences in age at onset, progression to renal replacement therapy, or hepatic complications were observed between males and females.

Collectively, this supports the concept that, whereas renal severity is largely driven by fibrocystin dosage, hepatic disease is influenced by additional age-dependent and modifying factors.

### 3.5. Family History of Adult-Onset Disease

**Figure 4:**
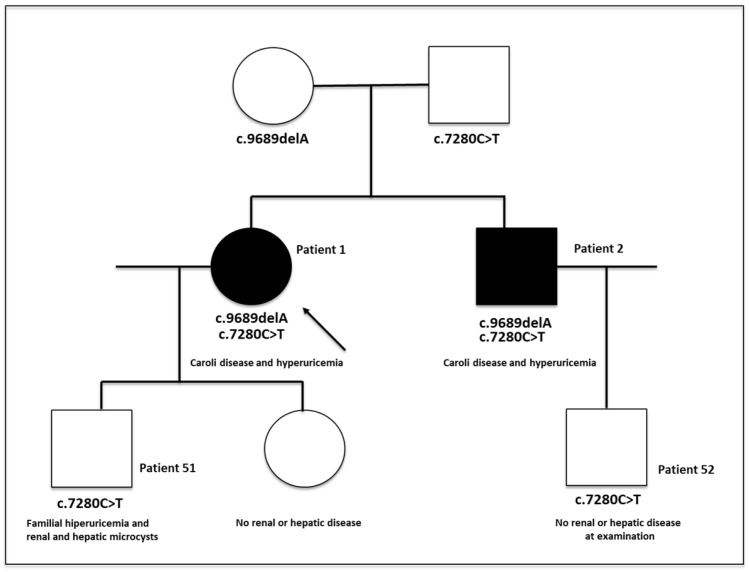
Pedigree of a family with adult-onset ARPKD.

This family (see Figure 4 for pedigree) is highly “unusual”, since no clinical features suggestive of ARPKD were observed until late adulthood, underscoring the marked phenotypic variability of the ARPKD disease spectrum. A definitive diagnosis was established using a targeted NGS panel-based approach.

#### 3.5.1. Patient 1 (Index)

A 64-year-old woman presented with an isolated episode of proteinuria at 15 years of age that resolved with conservative measures and without a definitive diagnosis. At 25 years, she experienced recurrent lower urinary tract infections, when mild right pyelocaliceal ectasia was detected in the absence of vesicoureteral reflux. Family history was notable for a father with nephrolithiasis, a mother with bone marrow dysplasia and cystic liver disease, and a paternal uncle with hyperuricemia and gout.

**Figure 5:**
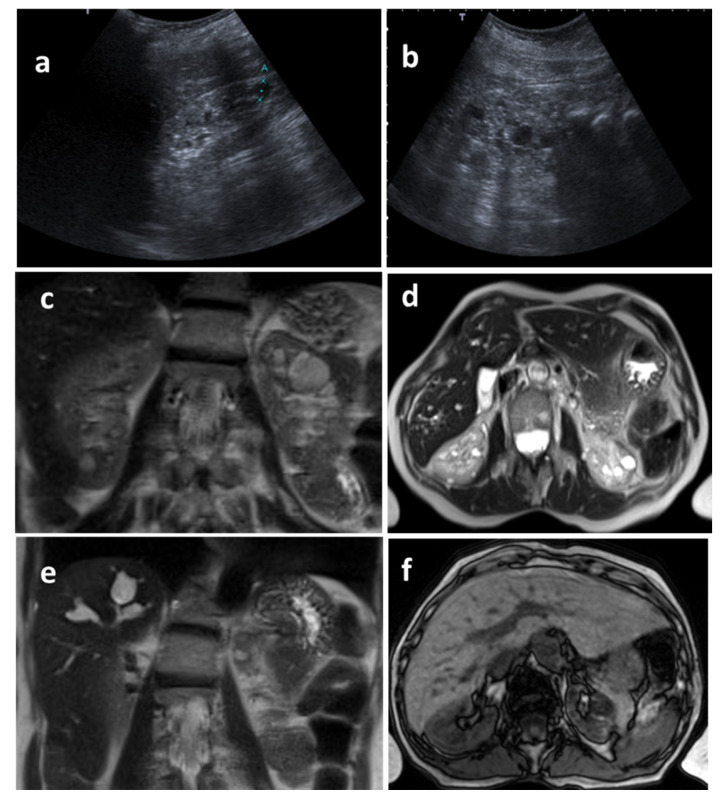
Image analysis of the index case. (**a**,**b**) Renal ultrasound, longitudinal view. The kidneys show preserved parenchymal thickness, although with markedly increased echogenicity and loss of corticomedullary differentiation. Multiple cystic images and parenchymal calcifications are observed. (**c**,**d**) MRI, coronal and axial sections, demonstrating multiple renal cysts resulting in an irregular renal contour. (**e**,**f**) MRI, coronal and axial sections, showing saccular and fusiform dilatations of the intrahepatic biliary tree.

She has been followed at our center since 2004 for kidney disease first identified in 2000 (age 48), characterized by mildly impaired renal function (serum creatinine 1.1–1.3 mg/dL; MDRD-eGFR 54–55 mL/min/1.73 m^2^) and asymptomatic hyperuricemia, without hypertension, microhematuria, or proteinuria. Renal ultrasound showed normal-sized kidneys with mildly hyperechogenic parenchyma and multiple medullary cysts with calcifications, suggestive of medullary sponge kidney. Renal function has remained stable, with normotension and pharmacologically controlled hyperuricemia. Abdominal MRI in 2008 revealed saccular dilatations of the intrahepatic bile ducts consistent with Caroli disease, while renal cystic involvement predominantly affected the medullary regions, with greater left kidney involvement. These findings remained stable on follow-up imaging, although progressive left renal volume reduction was noted (Figure 5).

Based on clinical, familial, and imaging features, the disease was initially classified as autosomal dominant medullary cystic kidney disease with Caroli disease; however, genetic testing for *UMOD*, *REN*, *HNF1B*, and *MUC1* genes was negative. Subsequent analysis identified two *PKHD1* variants: a pathogenic frameshift variant (c.9689delT, p.Asp3230Valfs34) and a pathogenic missense (c.7280T>C, p.Ile2427Thr), supporting a diagnosis within the ARPKD spectrum. The late-onset presentation was consistent with this diagnosis, whereas hyperuricemia was attributed to renal hypouricosuric hyperuricemia rather than the underlying ciliopathy.

#### 3.5.2. Patient 2

A 58-year-old man was referred to our clinic for evaluation of familial hyperuricemic nephropathy (brother of the index case). His past medical history included mild hyperuricemia (6.4–6.7 mg/dL) documented since 2000, asthma treated with occasional bronchodilator therapy, hiatal hernia, and tonsillectomy in childhood. With regard to his nephrourological history, in 1992 (at 34 years of age), he experienced two to three episodes of gross hematuria, with preserved renal function, absence of proteinuria, and no associated symptoms. Abdominal computed tomography revealed renal and hepatic cysts with precalyceal calcifications, leading to a diagnosis of possible Cacchi–Ricci disease (medullary sponge kidney), without indication for specific treatment. On follow-up magnetic resonance imaging performed in 2009, in addition to the renal and hepatic cysts, mild fusiform dilatation of the intrahepatic bile ducts was observed.

In 2011, he experienced an episode of renal colic associated with microhematuria, without documented stone passage, and in the same year underwent surgical repair of a hydrocele. Over subsequent years, he remained asymptomatic, with preserved renal function (serum creatinine 0.98 mg/dL; MDRD-estimated eGFR>60 mL/min/1.73 m^2^) and asymptomatic hyperuricemia with renal under-excretion of uric acid (fractional excretion of urate 4.61%), adequately controlled with pharmacological treatment (allopurinol 100 mg/day). Following the genetic diagnosis in his sister, targeted analysis of the *PKHD1* gene was performed, identifying the same variants (c.9689delT and c.7280T>C).

#### 3.5.3. Patient 51 (See Table S1)

A 34-year-old man was referred to our clinic in 2011 for evaluation of familial hyperuricemic nephropathy, given the suspected presence of this condition in his mother and maternal uncle. His medical history included asymptomatic hyperuricemia, without gout attacks and under adequate pharmacological control, as well as prior surgical repair of hypospadias with residual urethral stenosis requiring periodic dilatations. Renal function remained preserved (serum creatinine 1.0–1.1 mg/dL; MDRD-estimated GFR>60 mL/min/1.73 m^2^), and renal under-excretion of urate was documented (fractional excretion of urate 4%).

Renal and abdominal ultrasounds performed in 2002 and 2006 were unremarkable. In 2009, magnetic resonance imaging revealed no morphological abnormalities of either the liver or the kidneys, in contrast to the findings observed in his affected relatives. In view of the *PKHD1* variants identified in his relatives, targeted genetic analysis of this gene was performed by Sanger sequencing, which identified the same pathogenic missense variant in exon 46 (c.7280T>C), but not the frameshift pathogenic variant in exon 58 (c.9689delT).

#### 3.5.4. Patient 52 (See Table S1)

No renal or hepatic disease was established at the point of review the case.

## 4. Discussion

In this study, we present a comprehensive clinical and genetic characterization of a well-defined cohort of patients with ARPKD, integrating detailed longitudinal clinical data with *PKHD1* variant analysis.

### 4.1. Clinical Spectrum and Survival Compared with Published Cohorts

Overall survival in our cohort was substantially higher than that reported in historical ARPKD series, including the early cohorts described by Bergmann et al. [4,6,9,21,22] and the Günay-Aygun cohort [1,25], in which perinatal mortality typically ranged between 20% and 40% (Table S2). In contrast, only two neonatal deaths (4%) and three prenatal deaths (6%) were observed in our series, reflecting both survivor enrichment and major advances in neonatal respiratory and intensive care, currently. Notably, in addition, 36% of individuals survived into adulthood, consistent with recent national registries [8,12,13,23] that document a growing population of long-term ARPKD survivors (Table S2).

Renal involvement in our cohort was highly heterogeneous. While a subset of patients exhibited the classical neonatal presentation with markedly enlarged kidneys and early-onset chronic kidney disease, many others maintained a relatively preserved renal function into adolescence and adulthood. Ultimately, only 14% of patients required renal replacement therapy. This pattern is in line with contemporary ARPKD cohorts (Table S2), which indicate a shift from early progression to end-stage renal disease toward a phenotype characterized by chronic but often stable renal impairment. In contrast, hepatobiliary disease represented the major long-term morbidity in our cohort, affecting 74% of patients and leading to clinically significant portal hypertension in 34%. These frequencies are comparable to those reported in the Günay-Aygun’s cohort [1,25], but in our series, hepatic involvement was observed across all genotype classes. Importantly, several individuals displayed liver-predominant disease despite only mild renal manifestations, highlighting that ARPKD should no longer be viewed primarily as a renal disorder in contemporary clinical practice.

### 4.2. Genotype–Phenotype Correlations in ARPKD

Restricting analyses to molecularly confirmed ARPKD revealed a clear allelic-dose effect. The number of loss-of-function *PKHD1* alleles was the dominant determinant of renal and perinatal severity in ARPKD, with biallelic truncating variants conferring the highest risk of neonatal presentation and progression to end-stage kidney disease. This quantitative model is consistent with a threshold effect of fibrocystin deficiency and parallels observations in other recessive ciliopathies, where the nature and dosage of allelic variants modulate disease severity. For example, in nephronophthisis-related ciliopathies, two null alleles tend to drive more severe dysplastic phenotypes whereas the presence of at least one missense allele is associated with milder presentations, reflecting a burden-dependent severity gradient [26]. These findings are also consistent with previous reports (Table 5) and [27], indicating that truncating and canonical splice-site variants lead to greater disruption of fibrocystin function and more severe phenotypes. By contrast, patients carrying only missense variants exhibited a milder clinical course, with prolonged preservation of renal function and no requirement for kidney transplantation in this series, supporting the concept that at least some missense variants retain partial protein function and attenuate disease severity, as originally proposed by Rossetti and Harris [27]. Nevertheless, substantial phenotypic variability persisted, consistent with their observation that allelic class alone cannot fully explain clinical heterogeneity in ARPKD.

Notably, classification of variants based solely on ACMG pathogenicity criteria was less informative for phenotype stratification than classification based on a predicted functional effect, underscoring the importance of incorporating molecular consequence into genotype–phenotype analyses. Although prenatal abnormalities and the need for renal replacement therapy followed similar trends, limited sample size likely reduced statistical power for these outcomes. Overall, our data support the clinical utility of LoF allele burden as a practical and biologically meaningful predictor of disease severity, with implications for prognosis, genetic counseling, and longitudinal management.

In contrast, hepatic manifestations, including congenital hepatic fibrosis and portal hypertension, were common across all genotypic classes and showed little dependence on LoF burden. This dissociation suggests that distinct pathogenic mechanisms may underlie renal and biliary disease in *PKHD1*-associated disorders, explaining the frequent observation of severe hepatic disease in patients with relatively mild renal involvement. From a biological perspective, the absence of strong genotype–phenotype correlations for hepatic involvement may reflect the complex and multifactorial mechanisms underlying congenital hepatic fibrosis, including the contribution of modifier genes, epigenetic regulation, and environmental factors. In contrast to the kidney, where genetic burden appears to play a dominant role, hepatic disease severity is more closely related to age and duration of survival.

Our experimental findings appear to be in line with the recent study by Medaglia and colleagues [9], which highlights the marked discrepancy between predicted and observed prevalence in ARPKD, supporting the notion that many *PKHD1* variants, particularly variants of uncertain significance, do not cause the classical disease or exhibit reduced penetrance and/or very mild phenotypes. These variants would therefore be positioned at the hypomorphic end of the allelic and phenotypic continuum defined in our study, providing independent clinical validation of the fibrocystin dosage model. Indeed, our results provide a molecular explanation for the discrepancy between predicted and clinically observed ARPKD prevalence. While *PKHD1* harbors many rare missense variants, only a subset (those validated by segregation, apparently *de novo* allele occurrence, or strong functional evidence), are truly disease causing. Failure to apply such criteria leads to overestimation of ARPKD prevalence and spurious genotype–phenotype associations. Importantly, we further demonstrate that VUS-LP alleles should not be considered equivalent to generic VUS. When present *in trans* with a pathogenic or likely pathogenic allele and supported by segregation, VUS-LP variants function as disease-causing alleles, consistent with current ACMG guidance. The inclusion of these alleles (patients 25, 39, 40, and 43) did not compromise genotype–phenotype correlations and instead reinforced them, highlighting the importance of family-based validation in genes with extreme allelic heterogeneity, such as *PKHD1*.

Together, this longitudinal cohort reveals a clear genotype–phenotype signal driven by *PKHD1* LoF allele burden with respect to renal, pulmonary, and perinatal severity, alongside substantial hepatic morbidity that is largely independent of variant type or position. These findings are fully concordant with prior large ARPKD cohorts (Table 5 and  Table S2). Bergmann and colleagues demonstrated that *PKHD1* variant class strongly influences renal severity but not hepatic progression [6,9,20,21], while Adeva et al. [28] highlighted the age-dependent nature of congenital hepatic fibrosis and portal hypertension. Similarly, Günay-Aygun et al. [1] reported marked variability in hepatic outcomes without consistent associations with variant class or localization. Collectively, these data reinforce the concept that, unlike renal and pulmonary involvement, hepatic disease severity in ARPKD is not tightly determined by *PKHD1* genotype.

### 4.3. The Impact of NGS Targeted-Gene Panels in ARPKD

A major strength of this study is the systematic integration of NGS-based molecular diagnostics into the clinical evaluation of ARPKD. NGS panels enabled molecular characterization of patients with atypical or mild presentations, allowing identification of *PKHD1* variants that would not have been detected by phenotype-based testing alone, even when definitive molecular confirmation was not always possible. These findings support the emerging view of ARPKD as a broad phenotypic continuum shaped by allelic architecture and genetic modifiers.

However, this genomic expansion also creates a major interpretive challenge. *PKHD1* harbors hundreds of rare missense and splice-region variants, most of which are individually too rare to be confidently classified based on population data or in silico prediction alone. Without phase-aware and family-based interpretation, these variants are easily misclassified, leading to inflation of disease prevalence and spurious genotype–phenotype correlations, as recently highlighted by Medaglia et al. [9]. Our study directly addresses this challenge by distinguishing a molecularly confirmed ARPKD core from a broader *PKHD1*-associated variant spectrum using segregation-informed ACMG classification.

In this context, it is essential to differentiate between VUS and functional hypomorphism. Variants classified as VUS reflect a lack of sufficient evidence for or against pathogenicity. By contrast, hypomorphic alleles are defined here in a strictly functional sense as *PKHD1* variants for which genetic or biological data support partial preservation of protein activity, typically inferred from segregation with attenuated disease, recurrence in mild phenotypes, or published functional or transcript-level evidence. These two concepts are not synonymous. In a gene with extreme allelic heterogeneity such as *PKHD1*, the majority of rare missense variants are likely benign unless supported by such evidence, and we therefore deliberately avoid labeling unsolved VUS as hypomorphic.

Within this framework, NGS facilitates the identification of rare *PKHD1* variants, including a subset of hypomorphic alleles with functionally supported residual activity, which are enriched in patients with milder renal progression and delayed clinical presentation. These alleles contrast with truncating variants, which more consistently produce severe fibrocystin deficiency and drive neonatal or early childhood disease. Nevertheless, even among genetically confirmed ARPKD patients, substantial phenotypic variability persists, indicating that allelic class alone is insufficient to fully predict outcome and that modifier genes, epigenetic regulation, and developmental factors contribute to disease expression.

Taken together, our data and those of Medaglia et al. [9] converge on a unified model in which *PKHD1*-associated disease represents a continuum driven by fibrocystin dosage and allelic complexity. NGS does not merely broaden case detection; it necessitates a more rigorous, phase-aware, and function-based interpretation of variants in order to distinguish true ARPKD from a wider background of rare but largely benign *PKHD1* variation. This approach is essential to prevent overdiagnosis, to enable meaningful genotype–phenotype correlations, and to provide accurate prognostic and counseling information in the genomic era.

### 4.4. Strengths, Limitations, and Clinical Implications

The principal strength of this study lies in the integration of detailed longitudinal clinical data with contemporary NGS-based molecular analysis across a broad phenotypic spectrum of *PKHD1*-associated disease. The availability of long-term renal and hepatic outcomes, including renal replacement therapy, transplantation, pulmonary involvement, and survival, provides a robust framework for evaluating genotype–phenotype relationships beyond age at diagnosis alone.

Several limitations should nevertheless be acknowledged. Although 50 individuals carried biallelic *PKHD1* variants, not all met strict criteria for molecularly confirmed ARPKD, and a substantial proportion harbored at least one variant of uncertain significance (VUS), reflecting the high allelic heterogeneity of *PKHD1* and the challenges of variant interpretation. These cases were therefore analyzed within an expanded *PKHD1*-associated disease spectrum rather than as definitively affected ARPKD. In addition, functional validation of missense and VUS alleles was not available, meaning that although predicted molecular consequence (loss-of-function vs. non-loss-of-function) showed strong associations with renal severity, the pathogenicity of individual hypomorphic variants cannot be conclusively established. This limitation is inherent to most large NGS-based ARPKD cohorts and highlights the need for transcript-level, structural, and in vitro functional studies. Finally, incomplete segregation and phasing in some families limited definitive assessment of complex allelic configurations, including possible triallelic or modifier effects.

Despite these limitations, our findings underscore that NGS does not merely confirm classical ARPKD but reveals a continuum of *PKHD1*-associated hepatorenal disease, ranging from severe neonatal ARPKD to attenuated adult-onset phenotypes. Accurate interpretation of this spectrum is essential for genetic counseling, prenatal risk assessment, and long-term clinical management, particularly as genomic screening is increasingly applied in neonatal and adult nephrology settings.

### 4.5. Future Directions

Future research should prioritize several key directions. Longitudinal cohorts spanning childhood to adulthood are needed to define contemporary renal and hepatic disease trajectories as survival continues to improve. In parallel, the expansion of whole-genome sequencing in neonatal screening will enable more accurate estimation of the prevalence and penetrance of hypomorphic *PKHD1* alleles, informing variant interpretation and management of incidental findings. Emerging long-read sequencing and single-cell transcriptomic approaches will further clarify allelic complexity, splicing patterns, and tissue-specific consequences of *PKHD1* dysfunction. Finally, as more patients reach adulthood, integrated pediatric–adult care models and patient-reported outcomes will be essential to assess long-term disease burden and the impact of emerging antifibrotic and ciliopathy-targeted therapies. Collectively, these efforts will enhance diagnostic precision, prognostic stratification, and personalized care in ARPKD.

## 5. Conclusions

By integrating next-generation sequencing with segregation analysis and ACMG, we demonstrate that *PKHD1*-associated disease comprises a molecularly defined ARPKD core and a broader spectrum driven by variants of uncertain pathogenicity. In our cohort, 40 of 50 biallelic patients fulfilled criteria for molecularly confirmed ARPKD, while the remaining cases represented an expanded *PKHD1* variant spectrum. Within molecularly confirmed ARPKD, the number of *PKHD1* loss-of-function alleles is the primary determinant of renal and perinatal disease severity, with biallelic truncating variants conferring the highest risk of neonatal presentation and progression to renal failure. In contrast, hepatic involvement is highly prevalent across all genotypic classes and largely independent of truncating allele burden, highlighting divergent pathogenic mechanisms for kidney and biliary disease.

These findings refine diagnostic boundaries, improve genotype-based prognostication, and underscore the necessity of phase-aware and family-informed variant interpretation in genes with extreme allelic heterogeneity such as *PKHD1*. Together, they provide a robust framework for applying genomic data to clinical care in ARPKD.

## Figures and Tables

**Table 1:** Renal, hepatic, and pulmonary frequent clinical features observed in the cohort.

Feature	Patients	Frequency (%)
**Hepatic findings**		
Intrahepatic biliary duct dilatations (Caroli D.)	24/50	48
Hepatic fibrosis	13/50	26
Splenomegaly	13/50	26
Portal hypertension	12/50	24
Esophageal varices	5/50	10
Hepatomegaly	4/50	8
Multiple biliary cysts	2/50	4
Diffuse hepatic echogenicity alteration	2/50	4
Mild choledochal dilation	1/50	2
Chronic thrombocytopenia	1/50	2
Cholangitis	1/50	2
Cholestasis	1/50	2
No hepatic manifestations	17/50	34
**Renal findings**		
Bilateral renal cysts	18/50	36
Progressive eGFR decline	14/50	28
Increased echogenicity	14/50	28
Microcysts	10/50	20
Medullary cysts	9/50	18
Nephromegaly	7/50	14
Hypertension	5/50	10
Mild ectasia	5/50	10
Mild right pelvicalyceal dilatation	4/50	8
Increased renal volume	4/50	8
ESRD	4/50	8
Lithiasis	3/50	6
Hypouricemia	3/50	6
Moderate proteinuria	2/50	4
Prenatal renal dysplasia	1/50	2
Recurrent cystitis	1/50	2
Medullary sponge kidney	1/50	2
Bilateral medullar nephropathy	1/50	2
Absent renal manifestations	1/50	2
Non-available data	7/50	14
**Pulmonary findings**		
Neonatal pulmonary hypoplasia	4/50	8
Asthma	2/50	4
History of pulmonary hypoplasia	2/50	4
Neonatal pneumothorax	2/50	4
Bilateral pleural effusion	2/50	4
Transient pulmonary hypertension	1/50	2
Episodes of bronchospasm	1/50	2
Absent pulmonary findings	29/50	58
Non-available data	10/50	20

eGFR, estimated glomerular filtration rate; ESRD, end-stage renal disease. Extended clinical analysis is shown in Table S1.

**Table 2:** Distribution of biallelic *PKHD1* genotypes after ACMG reclassification and phase-aware resolution.

Patients	*PKHD1* (NM_138694.4)	Protein (p.)	Gender	Age at Diagnosis	Effect	Inheritance	ACMG
1	c.9689delA	p.Asp3230fs	Female	Adult	Frameshift	mat	P
	c.7280T>C	p.Ile2427Thr			Missense	pat	P
2	c.9689delA	p.Asp3230fs	Male	Adult	Frameshift	mat	P
	c.7280T>C	p.Ile2427Thr			Missense	pat	P
3	c.2710A>G	p.Thr904Ala	Female	Adult	Missense	?	VUS
	c.4105C>T	p.Arg1369Cys			Missense	?	VUS
4	c.5134G>C	p.Gly1712Arg	Female	Infant	Missense	mat	LP
	c.3474G>A	p.Trp1158fsTer			Nonsense	pat	P
5	c.1616T>C	p.Ile539Thr	Male	Infant	Missense	mat	LP
	c.1063G>T	p.Val355Phe			Missense	pat	LP
6	c.1937G>A	p.Trp646fsTer	Female	Adult	Nonsense	pat	P
	c.4427G>C	p.Cys1476Ser			Missense	mat	LP
7	c.9689delA	p.Asp3230fs	Female	Infant	Frameshift	pat	P
	c.2980C>T	p.Arg994Trp			Missense	mat	LP
8	c.8414T>A	p.Val2805Asp	Female	Adult	Missense	de novo	LP
	c.4870C>T	p.Arg1624Trp			Missense	mat	P
9	c.4292G>A	p.Cys1431Tyr	Male	Infant	Missense	pat	P
	c.4870C>T	p.Arg1624Trp			Missense	mat	P
10	c.8458T>G	p.Leu2820Val	Male	Adult	Missense	mat	LP
	c.2716-1G>T	p?			Non coding	pat	P
11	c.9107T>G	p.Val3036Gly	Female	Infant	Missense	pat	P
	c.9689delA	p.Asp3230fsTer			Frameshift	mat	P
12	c.5134G>C	p.Gly1712Arg	Female	Infant	Missense	pat	LP
	c.383delC	p.Thr128fsTer			Frameshift	mat	P
13	c.11215C>T	p.R3739W	Female	Infant	Missense	mat	LP
	c.5895dupA	p.Leu1966fsTer			Frameshift	pat	P
14	c.5134G>C	p.Gly1712Arg	Female	Infant	Missense	pat	LP
	c.107C>T	p.Thr36Met			Missense	mat	P
15	c.4118dupT	p.Met1373fsTer	Male	Infant	Frameshift	pat	P
	c.1486C>T	p.Gln496fsTer			Nonsense	mat	P
16	c.5825A>G	p.Asp1942Gly	Female	Infant	Missense	mat	LP
	c.6122-12G>A	p?			Splicing	pat	LP
	c.11506G>T	p.G3836fsTer			Nonsense	mat	P
17	c.10036T>C	p.Cys3346Arg	Female	Adult	Missense	mat	P
	c.8382C>G	p.Asp2794E			Missense	de novo	LP
18	c.5895dupA	p.Leu1966fsTer	Female	Adult	Frameshift	pat	P
	c.8492G>A	p.Arg2831Lys			Missense	de novo	LP
19	c.2280-1G>A	p?	Male	Infant	Non-coding	pat	P
	c.737T>C	p.Ile246Thr			Missense	mat	P
20	c.10856delA	p.Lys3619fsTer	Male	Infant	Frameshift	pat	P
	c.428A>G	p.Tyr143Cys			Missense	mat	LP
21	c.8388C>G	p.(Ser2796Arg)	Female	Infant	Missense	pat	LP
	c.458T>A	p.Ile153Lys			Missense	mat	LP
22	c.2414C>T	p.Pro805Leu	Female	Infant	Missense	pat	LP
	c.9530T>C	p.Ile3177Thr			Missense	mat	LP
23	c.6831delT	p.Tyr2277fsTer	Male	Infant	Nonsense	pat	P
	c.664A>G	p.Ile222Val			Missense	mat	P
24	c.10765C>T	p.Gln3589fsTer	Female	Infant	Nonsense	pat	P
	c.74T>A	p.Ile25Asn			Missense	mat	LP
25	c.5895dupA	p.Leu1966fsTer	Female	Infant	Frameshift	mat	P
	c.8492G>A	p.Arg2831Lys			Missense	pat	VUS-LP
26	c.3766delC mat	p.Gln1256fsTer	Male	Infant	Frameshift	mat	P
	c.3761C>G mat	p.Ala1254Gly			Missense	mat	VUS-LB
	c.5855A>C pat	p.Gln1952Pro			Missense	pat	LP
27	c.6383delT pat	p.Leu2128fsTer	Male	Infant	Frameshift	pat	P
	c.4292G>A mat	p.Cys1431Tyr			Missense	mat	P
	*PKD1*: C:8293C>T	p.Arg2765Cys			Missense	mat	hypomorphic
28	c.1736C>T	p.Thr579Met	Male	Adult	Missense	?	VUS-LB
	c.9689delA	p.Asp3230fs			Frameshift	?	P
29	c.4091A>G	p.Tyr1364Cys	Female	Infant	Missense	mat	LP
	c.5895dupA	p.Leu1966fsTer			Frameshift	pat	P
30	c.5895dupA	p.Leu1966fsTer	Male	Adult	Frameshift	mat	P
	c.6992T>A	p.Ile2331Lys			Missense	pat	P
31	c.9719G>A	p.Arg3240Gln	Female	Neonatal	Missense	mat	LP
	c.5895dupA	p.Leu1966fsTer			Frameshift	pat	P
	c.6992T>A	p.Ile2331Lys			Missense	mat	P
32	c.5895dupA	p.Leu1966fsTer	Female	Adult	Frameshift	mat	P
	c.4870C>T	p.Arg1624Trp			Missense	pat	P
33	c.9725G>A	p.Gly3242Asp	Female	Neonatal	Missense	pat	LP
	c.9719G>A	p.Arg3240Gln			Missense	mat	LP
34	c.8440+6T>C	p?	Male	Infant	Splicing	mat	LP
	c.8345G>C	p.Gly2782Ala			Missense	mat	VUS-LB
	c.5411delG	p.Arg1804fsTer			Frameshift	pat	P
35	c.9689delA	p.Asp3230fsTer	Female	Prenatal	Frameshift	pat	P
	c.778+3A>G	p.?			Non-coding	mat	LP
36	c.9689delA	p.Asp3230fsTer	Female	Prenatal	Frameshift	mat	P
	c.778+3A>G	p.?			Splicing	pat	LP
37	c.2264C>T	p.(Pro755Leu)	Male	Infant	Missense	pat	P
	c.2216C>T	p.Pro739Leu			Missense	mat	P
38	c.652G>A	p.Glu218Lys	Female	Infant	Missense	de novo	VUS-LP
	c.53-11C>T	p?			Splicing	de novo	VUS-LB
39	c.934C>T	p.Arg312Trp	Male	Infant	Missense	mat	VUS-LP
	c.107C>T	p.Thr36Met			Missense	pat	P
40	c.779-10_779-9insT	p?	Female	Infant	Non-coding	de novo	VUS-LP
	c.9689delA	p.Asp3230fsTer			Frameshift	mat	P
41	c.9998G>A	p.Arg3333Lys	Female	Adult	Missense	mat	LP
	c.5895dupA	p.Leu1966fster			Frameshift	pat	P
	*PKD2*:c.2398A>T	p.M800L			misense	pat	hypomorphic
42	c.9689delA	p.Asp3230fsTer	Male	Infant	Frameshift	mat	P
	c.2057A>C	p.His686Pro			Missense	pat	LP
43	c.10036T>C	p.Cys3346Arg	Male	Infant	Missense	pat	LP
	c.4304G>C	p.Ser1435Thr			Missense	mat	VUS-LP
44	c.5895dupA	p.Leu1966fsTer	Female	Adult	Frameshift	mat	P
	c.1234-10T>A	p.?			splicing?	de novo	VUS-LB
45	c.8581A>G	p.?	Female	Infant	Missense	mat	VUS modifier?
	c.2980C>T	p.Arg994Trp			Missense	pat	VUS-LP
	*PKD1*:c. 9338G>C	p.Gly3113Ala			Missense	mat	VUS-LB for PKD1
46	c.10585G>T	p.Glu3529Gln	Female	Prenatal	Nonsense	pat	LP
	c.1736C>T	p.Thr579Met			Missense	mat	VUS_LB
47	c.8108-16G>C	p?	Male	Infant	Non-coding	?	VUS_LB splicing?
	c.8798-19del	p?			Non-coding	?	VUS_LB splicing?
	*PKD2*:c.2411G>A	p.Ser804Asn			Missense	?	Hypomorphic
48	c.7744C>T	p.Pro2582Ser	Male	Adult	Missense	de novo	VUS_LB
	c.1736C>G	p.Thr579Arg			Missense	mat	VUS_LB
	c.2093G>A	p.Gly698Asp			Missense	pat	VUS_LB
49	c.8606C>A	p.Thr2869Lys	Male	Adult	Missense	mat	VUS, modifier?
	c.778+3A>G	p.?			Non-coding	de novo	LP
	c.3407A>G	p.Y1136C			misense	mat	VUS-LB
50	c.8345G>C	p.Gly2782Ala	Female	Infant	Missense	mat	VUS-LB
	c.5411delG	p.Arg1804fsTer			Frameshift	pat	P

ACMG, American College of Medical Geneticists; VUS, Variants of Unknown Significance; P, pathogenic; LP, likely pathogenic; LB, likely benign; mat, maternal; pat, paternal.

**Table 3:** Functional distribution of biallelic *PKHD1* genotypes.

Genotype Class	Definition	Patients (n)	Frequency (%)
LoF/LoF	Two truncating variants (frameshift, nonsense, or canonical splice)	17	34
LoF/non-LoF	One truncating + one missense or hypomorphic variant	14	28
non-LoF/non-LoF	Two missense or hypomorphic variants	5	10
Uncertain biallelic (≥1 VUS/LB)	At least one VUS, likely benign or conflicting allele	14	28
Total		50	100

The classification and distribution of the variants detected was according to the ACMG guidelines [19], and the functional predicted effect of the protein. LoF (nonsense, frameshift, or canonical splice-site) or non-LoF (missense and hypomorphic). ACMG, American College of Medical Geneticists; VUS, Variants of Unknown Significance; LB, likely benign.

**Table 4:** Genotype–phenotype correlations in molecularly confirmed ARPKD.

Variable	LoF/LoF	LoF/Non-LoF	Non-LoF/Non-LoF	*p*-Value
Neonatal/infantile onset	15 (88%)	9 (56%)	2 (29%)	<0.001 *
Childhood/adult onset	2 (12%)	7 (44%)	5 (71%)	<0.001 *
Renal replacement therapy (RRT)	11 (65%)	5 (31%)	0 (0%)	0.002 *
Portal hypertension	12 (71%)	10 (63%)	5 (71%)	>0.05
Congenital hepatic fibrosis (CHF)	14 (82%)	11 (69%)	5 (71%)	>0.05

The classification and distribution of the variants detected was according to the ACMG guidelines [19], and the functional predicted effect of the protein. LoF: nonsense, frameshift, or canonical splice-site) or non-LoF (missense and hypomorphic). ACMG, American College of Medical Geneticists. * *p* < 0.05, statistical significant result, Fisher’s exact test.

**Table 5:** Comparative genetic characteristics and genotype–phenotype correlations of major ARPKD cohorts reported in the literature.

Genetic Features	This Study	Bergmann et al. [4,6,21,22]	Günay–Aygun et al. [1,25]	Sharp et al. [8]	Burgmaier et al. [13]
Total number of variants reported	110	>750	200	120	563
Proportion of missense variants	~61%	35–45%	45–55%	~55%	~69%
Proportion of truncating variants (frameshift/nonsense)	~28%	50–60%	40–50%	35–40%	21%
Proportion of predicted splice-site variants	~11%	10–15%	10–15%	~10%	~6%
Missense-enriched genotype architecture	Yes (dominant)	No	Emerging	Strong	Yes (dominant)
Presence of recurrent hotspot alleles	Present (moderate frequency)	Frequent, historically highest	Present	Present	Present
Patients carrying ≥3 *PKHD1* variants	16% (10/50)	Very rare (pre-NGS era)	<5%	5–10%	2.6%
Biallelic truncating (null/null) variants	Very rare; associated with severe early disease or neonatal demise (few patients)	Strongly associated with perinatal lethality	Rare in survivors; lethal forms enriched in null/null	Rare; usually absent from long-term survivors	Rare; 4.3%
Missense–truncating genotypes	Most common combination; broad spectrum from severe neonatal to adult-onset disease	Frequent; severity variable but often moderate–severe	Common; associated with classic ARPKD but broad variability	Very common; significant variability	Common; moderate variability
Missense–missense genotypes	Frequent; observed in both mild and severe patients	Moderate; some milder courses	Common in moderate survivors	Frequent; associated with mild–moderate renal disease	Common; variable
Presence of hypomorphic alleles	Multiple; associated with survival into adulthood and milder renal trajectory	Documented but fewer	Documented	Documented	Not specifically Documented
Variant heterogeneity	High	Very high	High	High	High
Evidence for allelic complexity (triallelic or modifier effect)	Strong signal (16% ≥3 variants)	Not systematically evaluated	Discussed	Emerging	As limitation
Consistency of allele combinations with phenotype	Incomplete correlation	Incomplete correlation	Incomplete correlation	Incomplete correlation	Incomplete correlation
Association of genotype with renal severity	Weak overall; preserved function in many missense-rich genotypes	Strong correlation only for null/null patients	Moderate; wide phenotypic range within same genotypes	Phenotype highly variable despite allele types	Strong correlation for null/null patients
Association of genotype with hepatobiliary severity	Strong trend: hepatobiliary disease common across all genotype classes; liver-predominant forms noted	Hepatic disease increases with survival, genotype correlation weak	Hepatic severity largely independent of genotype	Significant hepatic involvement in survivors	Missense in AA 1838-2624 presented better hepatic outcome, aa. 2635-4074 the worst one
Portal hypertension and genotype	Observed across all genotype classes; no strong correlation		Very common independent of genotype	Variable	Not tightly genotype-linked
Patients with mild or “carrier-like” renal phenotype	Present even with biallelic variants	Rare	Reported	Reported	Not Reported
Patients with liver-predominant phenotype	Observed; may be linked to hypomorphic allele combinations	Rare in early severe cohorts	Increasingly documented	Reported	Discussed
Genotype predictiveness overall	Incomplete; phenotype cannot be reliably predicted from variant class	Incomplete	Incomplete	Incomplete	Incomplete

## Data Availability

The original contributions presented in this study are included in the article/Supplementary Material. Further inquiries can be directed to the corresponding authors.

## Supplementary Materials

The following supporting information can be downloaded at https://www.mdpi.com/article/10.3390/genes17020229/s1, Table S1: Whole clinical and genetic data in the cohort. Table S2: Comparison of clinical severity, survival, and genetic architecture among major ARPKD cohorts reported in the literature.
