# Supplementary material for: Next-Generation Sequencing Defines a Molecularly Confirmed ARPKD Core Within the Broader PKHD1-Associated Disease Spectrum
Source: Genes (Basel). 2026 Feb 11;17(2):229. doi: 10.3390/genes17020229 (PMC12940483; doi:10.3390/genes17020229)
Supplement: Supplementary file 1 [file genes-17-00229-s001.zip › Table S2.pdf]

Table S2. Comparison of clinical severity, survival, and genetic architecture among major ARPKD cohorts reported in the literature.

Feature	This Study	Bergmann et al.[4,6,21,22]	Günay –Aygun et al. [1, 25]	Sharp et al. [8]	Burgmaier et al. [13]
Cohort size	50	371 (meta-analysis)	73	42–65	304
Study population	Mixed pediatric + adult survivors	Mostly neonatal + early-childhood, many severe	Pediatric + adolescent, NIH longitudinal	Childhood + survivors	Mixed neonatal + survivors (young adults
Neonatal mortality	6%	25–40%	~20–25%	10–15%	Reported, but not isolated end-point is shown
Survival into adulthood	36%	Rare in early studies (<10%)	20–30%	25–35%	Commented, not reported
Renal replacement therapy (RRT)	14%	30–40%	25–30%	20–25%	Established, but not global number was showed
Liver transplantation	8%	5–10%	~10%	<10%	Established, but not global number was showed
Portal hypertension / varices	34%	20–30%	40–50%	25–30%	Not established
Hepatobiliary involvement (any)	74%	~60%	75–85%	60–70%	Frequent
Prenatal kidney findings	18%	70–80% (enriched for severe patients)	50–60%	40–50%	Nor established
Liver-predominant phenotype	Observed	Rare in early cohorts	Documented	Emerging	Emerging
Mild or subclinical renal phenotypes	Observed	Uncommon	Reported	Reported	Observed, especially Missense/Missense genotypes
Key novelty / distinguishing feature	Missense-enriched, long-term survivors with strong hepatobiliary morbidity; frequent multi-allelic findings	Largest early ARPKD genotype–phenotype definition	Most detailed hepatic natural history	Largest survivor-focused registry	The largest ARPKD genotype–phenotype cohort published to date. Genetic architecture dominated by missense variants (≈69%), rather than truncating variants.
